# COVID-19 and aerobic exercise: possible role of angiotensin converting enzyme 2

**DOI:** 10.1186/s13690-022-00983-3

**Published:** 2022-11-10

**Authors:** Haidar Djemai, Rami Hammad, Ibrahim M. Dabayebeh, Saleh Hammad, Abdellah Merzouk, Xavier Coumoul, Philippe Noirez

**Affiliations:** 1 Institute for Research in bioMedicine and Epidemiology of Sport (IRMES), National Institute of Sport, Expertise, and Performance (INSEP), 11 avenue du Tremblay, Paris, 75012 France; 2 INSERM UMR-S 1124, Environmental Toxicity, Therapeutic Targets, Cellular Signaling & Biomarkers (T3S), Université Paris Cité, 45 rue des Saints-Pères, Paris, 75006 France; 3 Département des Sciences de l’Activité Physique, Faculté des Sciences, Pavillon des Sciences Biologiques, UQAM, 141 avenue du Président-Kennedy, H2X 1Y4 Montréal, QC Canada; 4 Movement Science and Training, Faculty of Exercise Science, University of Jordan, Queen Rania St, PO Box 13617, 11942 Amman, Jordan; 5 Department of Physical and Health Education, Faculty of Educational Sciences, Al-Ahliyya Amman University, 19328 Amman, Jordan; 6 Physiological Adaptations to Exercise and Rehabilitation to Effort (APERE), Université de Picardie Jules Vernes, Allée Paschal Grousset, 3300, 80025 Amiens cedex 1, EA France; 7 Performance, Santé, Métrologie, Société (PSMS), UFR STAPS, Campus Moulin de la Housse, Université de Reims Champagne-Ardenne, Chemin des Rouliers, Reims, 51100 France

**Keywords:** COVID-19, SARS-CoV-2, Physical activity, Outdoor exercise

## Abstract

The emergence and circulation of a novel coronavirus (2019-nCoV)—Severe acute respiratory syndrome coronavirus 2 (SARS-CoV-2)—set off a global health crisis. SARS-CoV-2 spreads faster than its two ancestors, SARS-CoV and MERS-CoV. Several modes of transmission have been identified: via respiratory droplets, contact with infected people or contaminated surfaces, and potentially, bioaerosols. Various countries have taken preventive measures that may include partial or total lockdowns lasting weeks. The physical inactivity associated with lockdowns may promote cardiometabolic or other diseases, while physical activity may play a critical role in preventing them. Here we develop the hypothesis of the involvement of aerosols in the contamination process, the role of angiotensin converting enzyme 2 (ACE2), the potential benefits and harm of physical activity during lockdowns, and we suggest directions for future research.

## Highlights

• Physical inactivity during COVID-19 lockdowns may damage health.

• Risk of contamination by aerosols is higher during high-intensity urban outdoor exercise.

• Angiotensin converting enzyme 2 (ACE2) could play key role in SARS-CoV-2 transmission during exercise.

• How ACE2 serum concentration changes as function of exercise intensity and duration must be studied to elucidate role of this enzyme.

## Background

The global COVID-19 (previously 2019-nCOV) pandemic has not relented since it began in Wuhan, Hubei, China, in December 2019 [1]. The WHO has declared the new disease caused by SARS-CoV-2 a global public health emergency [1]. In 2021, even with the availability of treatment and vaccines [2], various countries continue to apply preventive measures that include school closings, restriction of non-food-related economic activity, and prohibition of unnecessary travel [3]. Such measures placed over a third of the world’s population under partial or total lockdown for several weeks during each wave of the pandemic [4, 5]. Lockdowns are characterized by more sedentary behavior [6], and considerable loss of muscle mass occurs after weeks without physical activity [3]. So et al. showed that during the 2003 SARS lockdown in China, lasting 7 weeks, levels of physical activity levels fell between 32% and 40% on average [7]. The WHO considers physical inactivity to be the fourth leading cause of mortality worldwide. It reports that 60–85% of people around the globe have sedentary lifestyles [8]. The sedentary behavior and physical inactivity associated with lockdowns might contribute to obesity and type 2 diabetes. Here we summarize the results of recent COVID-19 research and describe the health effects of lockdowns. We also discuss the potential benefits and harms of urban outdoor physical activity during COVID-19 lockdowns and the possible role played by angiotensin converting enzyme 2 (ACE2) in contamination.

### SARS-CoV-2 transmission and associated symptoms

Coronaviruses are primarily spread through close contact with other individuals [9]. Infection with SARS-CoV-2 usually occurs via contact with oral, nasal, or ocular mucous membranes, or by inhalation of droplets generated by coughing or sneezing [10]. Droplets can travel 1 to 2 m from their source [11, 12]. Aerosols are solid or liquid particles suspended and dispersed in the air. Some are atmospheric pollutants such as particulate matter of diameter ≤ 2.5 μm (PM_2.5_) or ≤ 10 μm (PM_10_). PM_2.5_ particles were thought to greatly contribute to COVID-19 infections in England [13]. Moreover, relationships between COVID-19 mortality and infectivity and air pollutant concentrations have recently been observed [13, 14].

After an infected expels the virus from their lungs and into the air, it may join with aerosols to form bioaerosols capable of traveling many meters [11, 12]. These bioaerosols can be easy inhaled, thereby entering the respiratory tract. Evidence suggests that most cases of SARS-CoV-1 and MERS-CoV were partly the result of aerosol inhalation [10, 15]. Epidemiological data show that transmissibility is higher for 2019-nCoV than for SARS-CoV or MERS-CoV [16]. Thus, the SARS-CoV-2 pandemic demands stricter measures to limit infection [17].

In some patients, initial clinical signs of this disease include a dry cough, breathing difficulties (dyspnea), and pneumonia [17–19]. In addition to pneumonia and dyspnea, 78% of patients with COVID-19 have been reported to have a fever (> 38 °C), and other symptoms are coughing, muscle pain, headache, stomachache, and diarrhea [20]. Loss of taste and smell has also been observed [21].

SARS-CoV-2 reportedly infects pulmonary alveolar epithelial cells by endocytosis [19]. It binds to the same ACE2 receptor as SARS-CoV, but with 10 to 20 times higher affinity [22]. Coronaviruses can enter cells expressing ACE2, replicate, spread, and cause disease. ACE2 is expressed on both type I and II alveolar epithelial cells, with expression dominant in the latter type (83%). ACE2 has also been shown to occur in other organs, such as the heart, esophagus, kidneys, bladder, and ileum [2, 23].

### Exercise and COVID-19 during pandemic

According to previous studies, endurance exercise increases ACE2 production through a complex mechanism involving beneficial systemic effects — e.g., increased angiotensin-(1–7) levels and decreased microRNA activity — as well as possible inflammatory responses [24–26]. ACE2 catalyzes the cleavage of angiotensin II into angiotensin-(1–7), a vasodilator that decreases blood pressure. Furthermore, exposure to SARS-CoV-2 apparently increases ACE2 receptor expression in the lungs [19, 27]. Although there is greater secretion of both types of ACE2 (i.e., as enzyme in plasma and as receptor in cardiovascular tissues) in patients with cardiovascular diseases, SARS-CoV-2 might also increase cardiovascular tissue expression of ACE2 in others [27, 28].

**Fig. 1:**
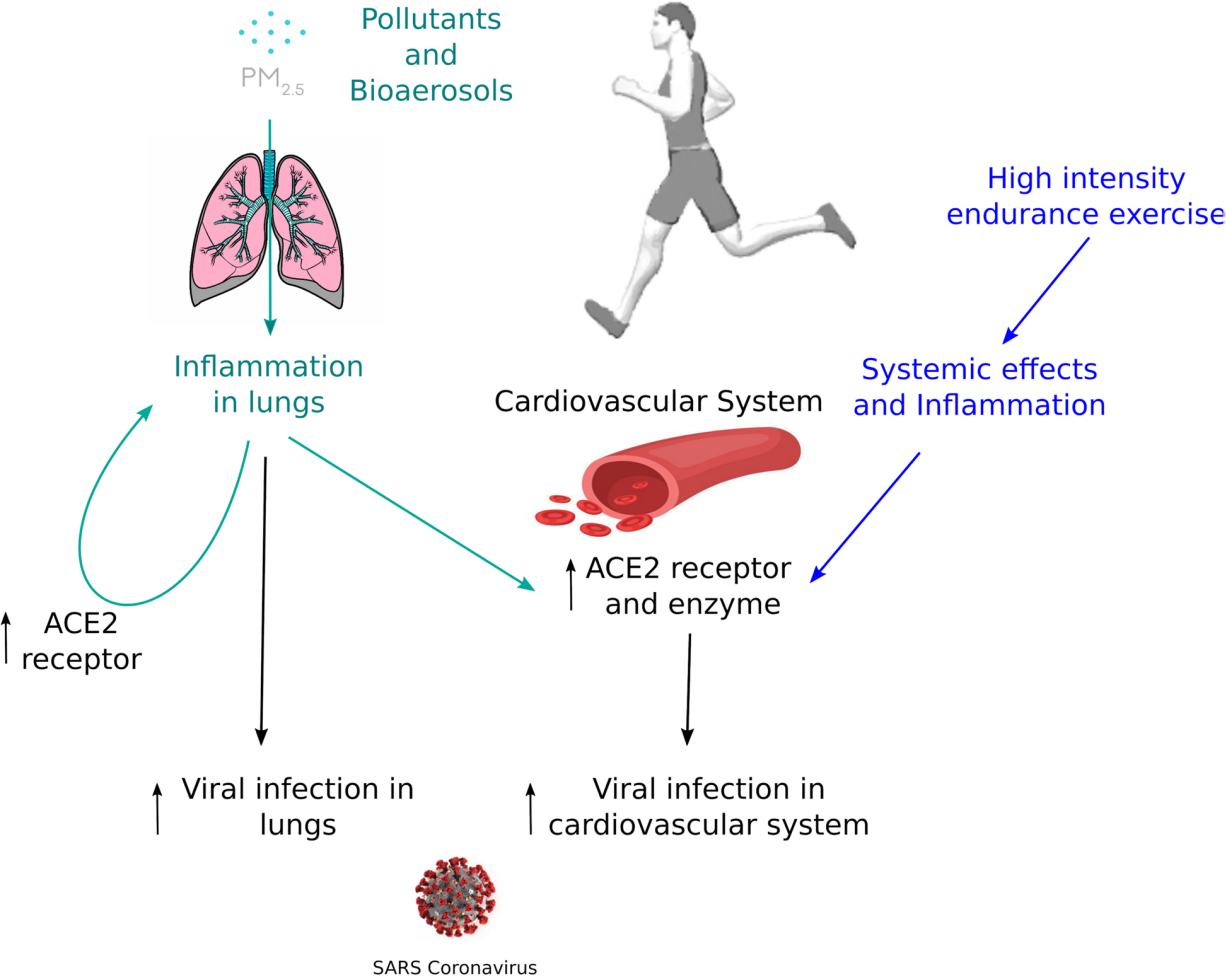
Possible mechanism of contamination by SARS-CoV-2 during endurance exercise within urban environment in presence of pollutants or bioaerosols. ACE2: angiotensin converting enzyme 2; PM_2.5_: fine particles (particulate matter) of diameter ≤ 2.5 μm

Endurance exercise is one of the main drivers of increased oxygen consumption (V̇O_2_) by working muscles: consumption increases linearly with exercise intensity. During endurance exercise, an incremental rise in O_2_ demand and CO_2_ production greatly increases ventilation, bringing athletes toward the ventilatory breakpoint [29]. This respiratory response might facilitate SARS-CoV-2 access to lung tissues. Increased ventilation induces greater recruitment of the pulmonary gas exchange surface to permit the passage of more oxygen into the blood [30, 31]. During aerobic exercise in big cities, greater doses of air pollutants can be inhaled [32]. As PM_2.5_ pollutant inhalation not only increases COVID-19 incidence [13] but also inflames lung tissue, it may lead to greater ACE2 receptor expression in alveolar epithelial cells [33]. Because SARS-CoV-2 can attach to fine particles, greater ventilation during exercise could entail rapid and massive transport of virions into the lungs, where they may bind to ACE2 receptors. Specifically, when running outdoors in an environment with a high concentration of fine airborne particles (i.e., pollutants or vapor droplets) and little air flow, large numbers of virions could be inhaled, subsequently affecting the cardiovascular system (Fig. 1) [4]. Further studies are needed to examine this possibility.

As suggested by So et al., it is reasonable to assume that SARS-CoV-2 may be found in sweat, just as it is in other bodily fluids [7]. The rise in body temperature during physical exercise can increase evaporation of sweat, leading to the formation of aerosolized viral particles capable of infecting others [7, 11, 12]. High-intensity endurance exercise in the streets of large cities during a pandemic could promote viral contagion and substantially heighten the risk of COVID-19-related cardiovascular injury. As the means of viral entry during exercise is not fully understood, avoidance of high-intensity outdoor physical activity in populated or polluted areas seems prudent [4].

## Hypothesis

The arguments presented above suggest physical activity during the COVID-19 pandemic promotes health. As lockdowns lasting multiple weeks may increase physical inactivity, sedentary behavior, obesity, and other health risks, regular physical activity may be of critical importance. We hypothesize that physical activity and air pollutants increase ACE2 receptor expression in the cardiovascular system and the lungs, respectively, which may in turn heighten the risk of SARS-CoV-2 infection (Fig. 1).

## Recommendations

During and after the pandemic, several studies have recommended physical exercise. Different protocols have been proposed combining aerobic and anaerobic exercises, indoors and outdoors, at different intensities and durations [34–36]. In the current situation our hypothesis suggests that high intensity outdoor physical activity requiring a high level of ventilation in urban environment is not recommended. This way, an add additional risk of viral infection via bioaerosols can be avoided.

## Conclusion

With the COVID-19 pandemic, among the prevention measures, a large portion of the world’s population was subjected to partial or total lockdowns for weeks. If physical activity is known to prevent cardiometabolic or other diseases, physical inactivity associated with lockdowns could play a critical role. Here we develop the hypothesis that the increase of angiotensin converting enzyme 2 (ACE2), caused by both physical activity and polluted urban air, may play a key role in the COVID-19 contamination process.

## Data Availability

Not applicable.

## Abbreviations

ACE2: Angiotensin converting enzyme 2
PM: Particulate matter
